# Ten inherited disorders in purebred dogs by functional breed groupings

**DOI:** 10.1186/s40575-015-0021-x

**Published:** 2015-07-11

**Authors:** A. M. Oberbauer, J. M. Belanger, T. Bellumori, D. L. Bannasch, T. R. Famula

**Affiliations:** Department of Animal Science, College of Agricultural and Environmental Sciences, University of California, Davis, One Shields Ave, Davis, CA 95616 USA; Department of Population Health and Reproduction, School of Veterinary Medicine, University of California, Davis, Davis, USA

**Keywords:** Purebred dog, Mixed-breed dog, Inherited disorders, Allele sharing

## Abstract

**Background:**

Analysis of 88,635 dogs seen at the University of California, Davis Veterinary Medical Teaching Hospital from 1995 to 2010 identified ten inherited conditions having greater prevalence within the purebred dog population as compared to the mixed-breed dog population: aortic stenosis, atopy/allergic dermatitis, gastric dilatation volvulus (GDV), early onset cataracts, dilated cardiomyopathy, elbow dysplasia, epilepsy, hypothyroidism, intervertebral disk disease (IVDD), and hepatic portosystemic shunt. The objective of the present study was to ascertain if disorders with higher prevalence in purebreds were restricted to particular breed group classifications within the purebred population, specifically the American Kennel Club breed grouping or groups with genomic similarities based upon allele sharing. For each disorder, healthy controls seen at the hospital during that same time period were matched for age, weight, and sex to each affected dog to determine risk of disease presentation in the purebred group as compared to that of the mixed-breed population. To enhance reliability of the analyses, sampling of matched healthy to affected dogs was repeated 50 times. For each comparison, the purebred subgroups to mixed-breed odds ratio was determined as was the mean *P* value used to test this ratio.

**Results:**

For aortic stenosis, GDV, early onset cataracts, dilated cardiomyopathy, elbow dysplasia, epilepsy, and portosystemic shunt, most purebred groups were not statistically distinct from the mixed-breed population with higher prevalence in purebreds restricted to distinct subsets of purebred dogs. The conditions of atopy/allergic dermatitis, hypothyroidism, and IVDD were more pervasive across the purebred population with many groups having higher prevalence than the mixed-breed population. The prevalence of IVDD in purebred terrier groups was statistically lower than that observed for mixed-breed dogs.

**Conclusions:**

The results offer an assessment of the distribution of inherited disorders within purebred dogs and illustrate how mixed-breed and subpopulations of purebred dogs do not differ statistically in prevalence for certain disorders. Some disorders appear linked to common ancestors providing insight into disease allele origin whereas others may be due to selection for common structural morphology. Knowledge of the origin of a condition may aid in reducing its prevalence in the dog population as a whole.

**Electronic supplementary material:**

The online version of this article (doi:10.1186/s40575-015-0021-x) contains supplementary material, which is available to authorized users.

## Lay summary

Although it is commonly assumed that purebred dogs are more prone to inherited (genetic) disorders than mixed-breed dogs, the data suggest that this distinction is not quite so categorical. In comparing the purebred dog population to the mixed-breed dog population for ten inherited conditions found at a higher frequency in purebred dogs, the current data indicate that risk for certain inherited disorders can be apportioned to specific purebred populations.

The inherited conditions of aortic stenosis (a narrowing above the aortic heart valve or the aortic valve itself), atopy/allergic dermatitis (skin allergies), gastric dilatation volvulus (bloat/stomach dilation), early onset cataracts (a clouding of the lens inside the eye), dilated cardiomyopathy (enlargement of the chambers of the heart and thinning of the muscle wall), elbow dysplasia (abnormal growth of tissues that leads to malformation and degeneration of the joint), epilepsy (brain seizures), hypothyroidism (underactive production of thyroid hormones), intervertebral disk disease (problems with the disks between the vertebrae of the spine leading to neurological problems), and hepatic portosystemic shunt (an abnormal blood circulation where blood is diverted around the liver rather than into it) are more prevalent in purebred dogs than in mixed-breed. Analyzing the prevalence of the conditions in subdivisions of the purebred population based upon the American Kennel Club classification of breed group or by relatedness at a DNA sequence level revealed that three conditions, atopy/allergic dermatitis, hypothyroidism, and intervertebral disk disease, were common across the purebred population with many of the purebred groups showing higher risk than the mixed-breed population. In contrast, for aortic stenosis, gastric dilation volvulus, early onset cataracts, dilated cardiomyopathy, elbow dysplasia, epilepsy, and portosystemic shunt, the prevalence in most purebred groups was not distinct from that seen in the mixed-breed population. These seven conditions showed higher risk only for certain subgroups of the purebred dog population.

This study suggests that subpopulations of the purebred dog population are more likely to exhibit certain inherited conditions while other subpopulations do not differ statistically from mixed-breed dogs in terms of how common these diseases are. Purebred groups with higher prevalence of particular disorders may reflect common ancestors or may be a consequence of selecting for a common structural morphology (e.g., shape or size). Understanding the genetic relationships behind a disease may provide new insight on how to best reduce its prevalence in the dog population as a whole.

## Background

The domestic dog is frequently cited as being an exceptional model for human inherited disorders due to the number of diseases identified, similarity in disease presentation, and population structure in the dog [1]. The ability to record health data for defined breeds has simultaneously permitted genetic dissection of particular disorders and bolstered the implicit assumption that purebred dogs are more prone to inherited disorders than mixed-breed dogs [2–5]. We recently have reported that for 13 inherited disorders, there were no statistically detectable differences in risk of disease presentation between the purebred and mixed-breed dog populations and one condition was more prevalent in mixed-breeds [6]. For ten other inherited conditions, the purebred dog population had greater prevalence than that seen in mixed-breeds: aortic stenosis, atopy/allergic dermatitis, gastric dilatation volvulus (GDV), early onset cataracts, dilated cardiomyopathy, elbow dysplasia, epilepsy, hypothyroidism, intervertebral disk disease (IVDD), and portosystemic shunt. It is known that many individual breeds show a higher than expected frequency of some disorders [7–10] suggesting that the higher prevalence of those disorders may be restricted to discrete subsets of the purebred dog population.

For example, Ubbink et al. [7] reported that certain disorders, such as elbow dysplasia and portosystemic shunt, are more likely to be found in dogs of related ancestral origin. Similarly, atopy/allergic dermatitis is found in greater prevalence in some breeds [11, 12] whereas other purebred dog breeds had equivalent prevalence to mixed-breed dogs [13]. Similar equivalences between populations were reported for other inherited conditions [9]. More clearly defining the populations most impacted by particular disorders is necessary to monitor alterations in incidence and seek constructive and effective mechanisms to reduce the burden of disease, thereby providing important knowledge for breeders, owners, researchers, and practitioners.

### Study objective

The objective of the present study was to ascertain if disorders having statistically higher prevalence in the purebred population could be attributed to particular purebred dog group classifications, such as American Kennel Club (AKC) breed groupings or groups having genomic similarities based upon allele sharing [14]. This study represents a further analysis of the database interrogated in the report by Bellumori et al., [6] in which the prevalence of inherited conditions were compared between purebred and mixed-breed dog populations seen at a veterinary teaching hospital over a 15-year period. Our analysis sought to describe the distribution of inherited disorders as a function of purebred categories when compared to the mixed-breed population for those ten inherited conditions determined to be more prevalent in the purebred population as a whole.

## Results and discussion

Previous studies indicate that some inherited disorders do not have significantly different prevalence across both the purebred and mixed-breed dog populations [6] which may represent ancient disease liability genes that preceded breed formation that are now distributed throughout the canine population as a whole or reflect recent purebred contributions to mixed-breed individuals. The present study identified specific breed groupings that contributed to disorders being more frequently observed in the purebred dog population. Disorders that were found to be more prevalent among subsets of the purebred population may be due to liability genes passed down from founding ancestors of related breeds, a consequence of selecting for a structural morphology that predisposes certain disorders, or the interplay between ancestral mutations and a morphological phenotype.

Overall disorder prevalence: The previous study [6] utilized all unique dogs seen at the University of California—Davis Veterinary Medical Teaching Hospital from January 1, 1995 through January 1, 2010. Because not all purebred dogs are recognized in the AKC breed groups, the total number of dogs assessed in the present study was 88,635. The number of mixed-breed dogs used in the analyses was 22,683. The number of purebreds able to be categorized into the AKC breed groupings and used in the analyses was 65,952 while the number of purebreds categorized based upon allele sharing was 55,353. The observed prevalence and estimated range of prevalence for each of the ten disorders are presented in Table 1. Inspection of the data indicates that prevalence for aortic stenosis, GDV, and dilated cardiomyopathy averaged less than 1 % in all dogs seen. In contrast, the average prevalence of early onset cataracts and IVDD exceeded 5 % across all dogs with the remaining disorders averaging 1–2 % prevalence. Estimating the prevalence of specific inherited disorders is an important component of assessing the welfare impact of disorders on the purebred dog population [15] and can provide insight into those disorders most amenable to improvement.

**Table 1:** Disorder counts, prevalence % (prev), and 95 % confidence interval (low and high) for inherited disorders between purebred dog categories and the mixed-breed dog population diagnosed over a 15-year period

	No. of normal	No. of affected	Prev	Low	High	No. of normal	No. of affected	Prev	Low	High	No. of normal	No. of affected	Prev	Low	High	No. of normal	No. of affected	Prev	Low	High	No. of normal	No. of affected	Prev	Low	High
	Aortic stenosis					Atopyl/allergic dermititis					Gastric dilatation volvulus					Early onset cataracts					Dilated cardiomyopathy				
AKC group																									
Mixed breed	22,649	34	0.15	0.10	0.20	22,437	246	1.08	0.95	1.21	22,638	45	0.20	0.14	0.26	21,767	916	4.04	3.78	4.30	22,647	36	0.16	0.11	0.21
Herding	9678	46	0.47	0.33	0.61	9604	120	1.23	1.01	1.45	9685	39	0.40	0.27	0.53	9377	347	3.57	3.20	3.94	9712	12	0.12	0.05	0.19
Hound	5331	13	0.24	0.11	0.37	5280	64	1.20	0.91	1.49	5329	15	0.28	0.14	0.42	5130	214	4.00	3.47	4.53	5317	27	0.51	0.32	0.70
Non-sporting	6491	25	0.38	0.23	0.53	6345	171	2.62	2.23	3.01	6489	27	0.41	0.25	0.57	5965	551	8.46	7.78	9.14	6508	8	0.12	0.03	0.21
Sporting	18,849	91	0.48	0.38	0.58	18,574	366	1.93	1.73	2.13	18,884	56	0.30	0.22	0.38	18,006	934	4.93	4.62	5.24	18,848	92	0.49	0.39	0.59
Terrier	4870	15	0.31	0.15	0.47	4730	155	3.17	2.68	3.66	4879	6	0.12	00.02	0.22	4557	328	6.71	6.01	7.41	4879	6	0.12	0.02	0.22
Toy	9773	25	0.26	0.16	0.36	9645	153	1.56	1.31	1.81	9795	3	0.03	0.00	0.06	8995	803	8.20	7.66	8.74	9794	4	0.04	0.00	0.08
Working	10,562	183	1.70	1.46	1.94	10,632	113	1.05	0.86	1.24	10,670	75	0.70	0.54	0.86	10,439	306	2.85	2.54	10538	207	1.93	1.67	2.19	
Haplotype group																									
Mixed breed	22,649	34	0.15	0.10	0.20	22,437	246	1.08	0.95	1.21	22,638	45	0.20	0.14	0.26	21,767	916	4.04	3.78	4.30	22,647	36	0.16	0.11	0.21
Ancient and spitz	2937	9	0.31	0.11	0.51	2910	36	1.22	0.82	1.62	2927	19	0.64	0.35	0.93	2795	151	5.13	4.33	5.93	2936	10	0.34	0.13	0.55
Herding	4568	11	0.24	0.10	0.38	4524	55	1.20	0.88	1.52	4573	6	0.13	0.03	0.23	4383	196	4.28	3.69	4.87	4577	2	0.04	−0.02	0.10
Mastiff-like	5443	105	1.89	1.53	2.25	5431	117	2.11	1.73	2.49	5534	14	0.25	0.12	0.38	5278	270	4.87	4.30	5.44	5494	54	0.97	.71	1.23
Retriever	18,181	166	0.90	0.75	1.04	18,032	315	1.72	1.53	1.91	18,269	78	0.43	0.34	0.52	17,783	564	3.07	2.82	3.32	18,231	116	0.63	0.52	0.74
Scent hound	3738	4	0.11	0.01	0.21	3692	50	1.34	0.97	1.71	3738	4	0.11	0.01	0.21	3558	184	4.92	4.23	5.61	3740	2	0.05	−0.02	0.12
Sight hound	757	4	0.53	0.02	1.04	757	4	0.53	0.02	1.04	755	6	0.76	0.16	1.42	744	17	2.23	1.18	3.28	745	15	1.97	0.98	2.96
Small terrier	2532	2	0.08	–0.08	0.19	2438	96	3.79	3.05	4.53	2533	1	0.04	–0.04	0.12	2369	165	6.51	5.55	7.47	5232	2	0.08	–0.03	0.19
Spaniel	4309	6	0.14	0.03	0.25	4239	76	1.76	1.37	2.15	4305	10	0.23	0.09	0.37	3884	431	9.99	9.10	10.88	4274	41	0.95	0.66	1.24
Toy	5524	18	0.32	0.17	0.47	5461	81	1.46	1.14	1.78	5541	1	0.02	−0.02	0.06	5204	338	6.10	5.47	6.73	5540	2	0.04	−0.04	0.09
Working group 1	1911	0	0.00	0.00	0.00	1883	28	1.47	0.93	2.01	1887	14	0.73	0.35	1.11	1666	245	12.82	11.32	14.32	1908	3	0.16	−0.02	0.34
Working group 2	5088	28	0.55	0.35	0.75	5044	72	1.41	1.09	1.73	5072	44	0.86	0.61	1.11	4981	135	2.64	2.20	3.08	5034	82	1.60	1.26	1.94
	Elbow dysplasia					Epilepsy					Hypothyroidism					Intervertebral disk disease					Portosystemic shunt				
AKC group																									
Mixed breed	22,479	204	0.90	0.78	1.02	22,477	206	0.91	0.79	1.03	22,334	349	1.54	1.38	1.70	21,679	1004	4.43	4.16	4.70	22,604	79	0.35	0.27	0.43
Herding	9555	169	1.74	1.48	2.00	9574	150	1.54	1.30	1.78	9553	171	1.76	1.50	2.02	9057	667	6.86	6.36	7.36	9695	29	0.30	0.19	0.41
Hound	5324	20	0.37	0.21	0.53	5270	74	1.38	1.07	1.69	5244	100	1.87	1.51	2.23	4278	1066	19.95	18.88	21.02	5313	31	0.58	0.38	0.79
Non-sporting	6481	35	054	0.36	0.72	6422	94	1.44	1.15	1.73	6387	129	1.98	1.64	2.32	6112	404	6.20	5.61	6.79	6488	28	0.43	0.27	0.59
Sporting	18,447	493	2.60	2.37	2.83	18,667	273	1.44	1.27	1.61	18,409	531	2.80	2.56	3.04	18,176	764	4.03	3.75	4.31	18,864	76	0.40	0.31	0.49
Terrier	4862	23	0.47	0.28	0.66	4844	41	0.84	0.58	1.10	4809	76	1.56	1.21	1.91	4711	174	3.56	3.04	4.08	4816	69	1.41	1.08	1.74
Toy	9791	7	0.07	0.02	0.12	9674	124	1.27	1.05	1.49	9675	123	1.23	1.04	1.48	9221	577	5.89	5.42	6.36	9412	386	3.94	3.55	4.33
Working	10,345	400	3.72	3.36	3.36	4.08	10,669	76	0.71	0.55	0.87	10438	307	2.86	2.54	3.18	10,062	683	6.36	5.90	10,712	33	0.31	0.21	0.41
Haplotype group																									
Mixed breed	22,479	204	0.90	0.78	1.02	22,477	206	0.91	0.79	1.03	22,334	349	1.54	1.38	1.70	21,679	1004	4.43	4.16	4.70	22,604	79	0.35	0.27	0.43
Ancient and spitz	2916	30	1.02	0.66	1.38	2906	40	1.36	0.94	1.78	2848	98	3.33	2.68	3.98	2810	136	4.62	3.86	5.38	2937	9	0.31	0.11	0.51
Herding	4553	26	0.57	0.35	0.79	4487	92	2.01	1.60	2.42	4477	102	2.23	1.80	2.66	4344	235	5.13	4.49	5.77	4555	24	0.52	0.31	0.73
Mastiff-like	5482	66	1.19	0.09	1.48	5496	52	0.94	0.69	1.19	5471	77	1.39	1.08	1.70	5263	285	5.14	4.56	5.72	5524	24	0.43	0.26	0.60
Retriever	17,557	790	4.31	4.02	4.60	18,145	202	1.10	0.95	1.25	17,876	471	2.57	2.34	2.80	17,522	825	4.50	4.20	4.80	18,279	68	0.37	0.28	0.45
Scent hound	3733	9	0.24	0.08	0.40	3686	56	1.50	1.11	1.89	3681	61	1.63	1.22	2.04	2760	982	26.24	24.83	27.65	3718	24	0.64	0.38	0.90
Sight hound	760	1	0.13	−0.13	0.39	750	11	1.45	0.60	2.30	748	13	1.71	0.79	2.63	706	55	7.23	5.39	9.07	755	6	0.79	0.16	1.42
Small terrier	2532	2	0.08	−0.03	0.19	2513	21	0.83	0.48	1.18	2503	31	1.22	0.79	1.65	2463	71	2.80	2.16	3.44	2367	167	6.59	5.62	7.56
Spaniel	4302	13	0.30	0.14	0.46	4235	80	1.85	1.45	2.25	4191	124	2.87	2.37	3.37	4112	202	4.68	4.05	5.31	4299	16	0.37	0.19	0.55
Toy	5539	3	0.05	−0.01	0.11	5473	69	1.25	0.96	1.54	5477	65	1.17	0.89	1.45	5153	389	7.02	6.35	7.69	5383	159	2.87	2.43	3.31
Working group 1	1908	3	0.16	−0.02	0.34	1869	42	2.20	1.54	2.86	1882	29	1.52	0.97	2.07	1819	92	4.81	3.85	5.77	1885	26	1.36	0.84	1.88
Working group 2	4992	124	2.42	2.00	2.84	5055	61	1.19	0.89	1.49	4998	118	2.31	1.90	2.72	4594	522	10.20	9.37	11.03	5107	9	0.18	0.07	0.29

Dog breed category: In AKC breed group categories, the terrier and toy groups showed greater probability of presenting with two of the disorders when compared to mixed-breed dogs (Tables 2 and 3). Herding and hound groups showed higher probabilities for four, non-sporting in five, working in six, and sporting in seven disorders as compared to the mixed-breed population. When considering the genomic similarities of the breeds based on haplotype sharing, ancient and spitz, herding, and sight hounds showed greater probability for one of the disorders when compared to mixed-breed dogs whereas scent hound, small terrier, and toy breeds showed higher probability in two, mastiff-like, working group 1, and spaniel in four, and retrievers and working group 2 for six of the inherited disorders.

**Table 2:** Odds ratios of inherited disorders between purebred dog categories and the mixed-breed dog population diagnosed over a 15-year period

	OR (CI)	*P* value	No.	OR (CI)	*P* value	No.	OR (CI)	*P* value	No.	OR (CI)	*P* value	No.	OR (CI)	*P* value	No.
	Aortic stenosis			Atopy/allergic dermatitis			Gastric dilatation volvulus			Early onset cataracts			Dilated cardiomyopathy		
AKC Group															
Herding	2.25(1.25–4.05)	0.02	44	1.06(0.79–1.43)	0.58	0	1.63(0.83–3.23)	0.23	9	0.99(0.82–1.20)	0.67	0	0.73(0.34–1.56)	0.45	0
Hound	1.50(0.66–3.45)	0.41	4	1.20(0.82–1.76)	0.14	2	2.72(1.01–7.34)	0.09	37	0.83(0.66–1.03)	0.13	15	4.98(2.29–10.83)	*0.00*	50
Non-sporting	2.00(0.98–4.08)	0.09	26	2.49(1.83–3.38)	*0.00*	50	3.45(1.51–7.88)	0.01	46	1.72(1.44–2.06)	*0.00*	50	0.91(0.34–2.39)	0.63	0
Sporting	2.10(1.25–3.52)	0.02	45	1.71(1.35–2.16)	*0.00*	50	1.20(0.67–2.14)	0.52	0	1.61(1.38–1.87)	*0.00*	50	2.40(1.45–3.97)	*0.00*	50
Terrier	2.02(0.84–4.87)	0.19	13	2.98(2.11–4.21)	*0.00*	50	1.33(0.33–5.38)	0.60	0	0.95(0.78–1.15)	0.52	0	0.86(0.24–3.08)	0.58	0
Toy	1.81(0.81–4.09)	0.22	7	1.76(1.23–2.47)	*0.01*	46	0.90(0.14–5.73)	0.20	0	1.16(0.98–1.37)	0.13	21	0.66(0.14–3.07)	0.56	0
Working	6.26(3.74–10.45)	0.00	50	0.96(0.71–1.31)	0.65	0	2.45(1.34–4.48)	0.01	48	1.23(1.00–1.52)	0.11	24	11.60(6.87–19.50)	*0.00*	50
Haplotype group															
Ancient and spitz	1.49(0.58–3.79)	0.44	0	1.15(0.71–1.85)	0.56	1	2.27(0.87–5.91)	0.15	16	1.35(1.02–1.79)	0.08	26	1.88(0.77–4.60)	0.25	6
Herding	1.17(0.48–2.89)	0.59	0	0.97(0.65–1.47)	0.61	0	0.29(0.06–1.42)	0.16	11	0.91(0.72–1.15)	0.45	0	0.26(0.06–1.19)	0.09	12
Mastiff-like	8.30(4.59–15.02)	*0.00*	50	2.11(1.50–2.97)	*0.00*	50	1.42(0.58–3.50)	0.49	4	1.44(1.15–1.81)	*0.01*	48	7.24(3.76–13.95)	*0.00*	50
Retriever	3.76(2.27–6.23)	*0.00*	50	(1.22–2.02)	*0.01*	49	1.63(0.93–2.85)	1.15	16	1.26(1.05–1.51)	*0.05*	38	3.22(.195–5.32)	*0.00*	50
Scent hound	0.92(0.24–3.53)	0.63	0	1.46(0.92–2.30)	0.18	16	2.27(0.38–13.49)	0.43	0	0.93(0.73–1.19)	0.54	0	0.90(0.16–4.94)	0.68	0
Sight hound	2.77(0.61–12.55)	0.28	3	0.47(0.15–1.48)	0.22	3	4.16(0.81–21.45)	0.16	15	0.55(0.28–1.08)	0.13	19	14.72(3.88–55.84)	*0.00*	50
Small terrier	0.51(0.09–3.03)	0.45	0	3.86(2.45–6.08)	0.00	50	0.71(0.04–13.40)	0.72	0	0.87(0.67–1.12)	0.33	2	1.95(0.22–17.08)	0.62	0
Spaniel	0.57(0.17–1.93)	0.38	0	1.57(1.07–2.32)	0.06	35	1.94(0.65–5.74)	0.32	3	1.88(1.54–2.30)	*0.00*	50	6.01(2.72–13.28)	*0.00*	50
Toy	1.91(0.74–4.93)	0.25	8	1.62(1.07–2.44)	0.05	35	0.52(0.03–8.73)	0.63	0	0.88(0.72–1.09)	0.30	6	1.13(0.15–8.26)	0.62	0
Working group 1	0.56(0.04–9.54)	0.98	0	1.75(0.98–3.13)	0.12	21	80.03(1.93–13.45)	*0.04*	47	2.17(1.65–2.85)	0.00	50	1.78(0.38–8.37)	0.53	0
Working group 2	2.45(1.24–4.)	0.03	43	1.26(.087–1.83)	0.29	5	3.40(1.65–7.04)	*0.01*	48	0.89(0.67–1.18)	0.43	2	7.42(4.08–13.50)	*0.00*	50
	Elbow dysplasia			Epilepsy			Hypothyroidism			IVDD			Portosystemic shunt		
AKC group															
Herding	1.65(1.23–2.21)	*0.00*	48	1.57(1.14–2–2.16)	*0.02*	44	1.11(0.86–1.42)	0.46	1	1.53(1.31–1.79)	*0.00*	50	0.86(0.49–1.53)	0.56	0
Hound	0.76(0.43–1.43)	0.40	2	1.65(1.09–2.50)	*0.05*	38	1.53(1.09–2.13)	*0.04*	37	4.54(3.82–5.40)	*0.00*	50	1.12(0.62–1.93)	0.59	0
Non-sporting	0.72(0.45–1.16)	0.23	7	1.49(1.02–2.17)	0.08	31	1.43(1.06–1.92)	*0.04*	37	1.31(1.10–1.56)	*0.01*	48	0.80(0.47–1.38)	0.46	3
Sporting	2.25(1.79–2.82)	*0.00*	50	1.51(1.15–1.98)	*0.02*	45	1.69(1.40–2.06)	*0.00*	50	0.93(0.81–1.07)	0.37	2	1.43(0.90–2.28)	0.21	14
Terrier	1.37(0.74–2.55)	0.37	1	0.93(0.58–1.50)	0.56	1	1.18(0.82–1.70)	0.43	4	0.57(0.46–0.71)	*0.00*	50	2.00(1.27–3.13)	*0.01*	46
Toy	0.67(0.19–2–40)	0.52	1	1.50(1.01–2.24)	0.09	26	1.31(0.91–1.88)	0.21	11	0.99(0.84–1.18)	0.65	0	4.15(2.89–5.96)	*0.00*	50
Working	2.89(2.25–3.70)	0.00	50	0.80(0.55–1.15)	0.28	5	2.17(1.72–2.74)	*0.00*	50	1.78(1.51–2.09)	*0.00*	50	1.00(0.56–1.80)	0.68	0
Haplotype group															
Ancient and spitz	1.07(0.64–1.76)	0.64	0	1.30(.077–2.19)	0.41	3	2.00(1.41–2.84)	*0.00*	50	0.97(0.75–1.27)	0.64	0	1.24(0.47–3.21)	0.59	0
Herding	0.71(0.42–1.20)	0.26	3	1.97(1.32–2.92)	*0.00*	49	1.30(0.93–1.79)	0.18	16	1.01(0.82–1.25)	0.66	0	1.06(0.56–2.00)	0.64	0
Mastiff-like	1.17(0.80–1.72)	0.46	2	1.15(0.75–1.78)	0.48	1	1.21(0.86–1.71)	0.37	6	1.21(0.99–1.48)	1.10	23	0.81(0.46–1.43)	48	0
Retriever	3.24(2.60–4.03)	*0.00*	50	1.21(0.90–1.63)	0.25	5	1.62(1.32–1.99)	*0.00*	50	1.11(0.96–1.29)	0.25	12	1.41(0.85–2.35)	0.25	4
Scent hound	1.00(0.40–2.49)	0.65	0	1.99(1.22–3.25)	*0.02*	45	1.61(1.05–2.47)	0.06	28	6.33(5.22–7.68)	*0.00*	50	1.04(0.57–1.87)	0.68	0
Sight hound	0.15(0.02–1.13)	0.08	17	1.51(0.59–3.88)	0.45	2	1.11(0.51–2.42)	0.62	0	1.39(0.91–2.13)	0.20	9	1.89(0.55–6.58)	0.36	0
Small terrier	0.28(0.02–3.66)	0.44	1	0.93(0.48–1.79)	0.62	0	1.23(0.70–2.17)	0.51	2	0.51(0.38–0.70)	*0.00*	50	6.90(4.36–10.93)	0.00	50
Spaniel	0.57(0.29–1.11)	0.15	16	1.81(1.20–2.73)	0.02	46	1.76(1.27–2.43)	*0.01*	49	0.88(0.71–1.09)	0.28	2	0.76(0.40–1.55)	0.48	0
Toy	0.40(0.6–2.80)	0.37	0	1.42(0.89–2.26)	0.22	14	1.22(0.77–1.93)	0.44	4	1.24(1.51)	0.07	32	2.92(1.95–4.38)	*0.00*	50
Working group 1	0.30(0.08–1.06)	0.09	22	2.54(1.40–4.61)	*0.01*	48	1.26(0.72–2.20)	0.47	2	1.19(0.88–1.61)	0.33	3	2.23(1.14–4.37)	*0.05*	35
Working group 2	1.88(1.35–2.30)	*0.00*	50	1.40(0.91–2.15)	0.20	10	1.49(1.09–2.02)	*0.04*	40	2.51(2.07–3.03)	*0.00*	50	0.74(0.29–1.88)	0.53	0

**Table 3:** Summary illustration of statistically different probabilities of inherited disorders between different purebred dog categories and the mixed-breed dog population

Breed category	Aortic stenosis	Atopy/Allergic dermatitis	Gastric dilatation volvulus	Early onset cataracts	Dilated cardio-myopathy	Elbow dysplasia	Epilepsy	Hypo-thyroidism	IVDD	Porto-systemic shunt
AKC										
Herding	↑	↔	↔	↔	↔	↑	↑	↔	↑	↔
Hound	↔	↔	↔	↔	↑	↔	↑	↑	↑	↔
Non-sporting	↔	↑	↑	↑	↔	↔	↔	↑	↑	↔
Sporting	↑	↑	↔	↑	↑	↑	↑	↑	↔	↔
Terrier	↔	↑	↔	↔	↔	↔	↔	↔	↓	↑
Toy	↔	↑	↔	↔	↔	↔	↔	↔	↔	↑
Working	↑	↔	↑	↔	↑	↑	↔	↑	↑	↔
Haplotype Share										
Ancient & Spitz	↔	↔	↔	↔	↔	↔	↔	↑	↔	↔
Herding	↔	↔	↔	↔	↔	↔	↑	↔	↔	↔
Mastiff-like	↑	↑	↔	↑	↑	↔	↔	↔	↔	↔
Retriever	↑	↑	↔	↑	↑	↑	↔	↑	↔	↔
Scent hound	↔	↔	↔	↔	↔	↔	↑	↔	↑	↔
Sight hound	↔	↔	↔	↔	↑	↔	↔	↔	↔	↔
Small terrier	↔	↑	↔	↔	↔	↔	↔	↔	↓	↑
Spaniel	↔	↔	↔	↑	↑	↔	↑	↑	↔	↔
Toy	↔	↑	↔	↔	↔	↔	↔	↔	↔	↑
Working 1	↔	↔	↑	↑	↔	↔	↑	↔	↔	↑
Working 2	↑	↔	↑	↔	↑	↑	↔	↑	↑	↔

Upward arrows indicate a significant increased risk of presentation whereas downward arrows indicate a reduced risk of the condition relative to mixed-breed dogs. Double headed sideways arrows indicate no statistical difference in disorder presentation

Data presented as mean OR (with 95 % CI) of the purebred group relative to mixed-breed dogs, mean *P* value of the matched control sampling sets, and the number of times (of 50) that those matched control sampling sets indicated a significant difference in probability that mixed-breed and purebred categories differed in prevalence of each condition (denoted in italics)

Red upward arrows indicate a significant increased risk of presentation whereas green downward arrows indicate a reduced risk of the condition relative to mixed-breed dogs. Double-headed sideway arrows indicate no statistical difference in disorder presentation

It is important to note that for some AKC groupings, disparate breeds may be associated based upon morphological or functional similarities rather than genetic relatedness. Similarly, the haplotype sharing categories may not be intuitive to individuals more familiar with the AKC breed groupings. For instance, in AKC, the German shepherd dog is categorized as a member of the herding group whereas in the haplotype sharing scenario, German shepherd dogs are associated with the working dog 2 category, sharing genomic similarities to the Doberman pinscher and Portuguese water dog. Standard poodles are in the AKC non-sporting group but in the working dog 1 haplotype association scheme, Havanese are in the AKC toy group yet are categorized as haplotype working dog 1, and the AKC sporting group includes retriever and spaniel breeds while the haplotype sharing approach splits those into two distinct groups. Dissimilar associations in the different categorization schemes may add complexity to interpretation while in some cases, it may provide clarity as to the significant breed contributions to particular disorders as detailed below.

Disorder by breed groupings: Defining the lineage associations for particular disorders may provide approaches to both breeding strategies and therapeutic interventions. Despite the large number of health records analyzed, there was insufficient representation of every individual breed to define prevalence or ascribe risk in particular purebred breeds. Nevertheless, utilizing functional and genomic relationships yielded meaningful associations of risk for purebred groupings. For disorders found in greater prevalence within the purebred population [6], subdividing the purebred population into discrete categories revealed that some disorders did not statistically differ in proportions between purebred subgrouping and the mixed-breed population (Tables 2 and 3). A higher probability of presenting with a disorder was often restricted to certain purebred subsets or haplotype associations rather than the entire purebred population.

Aortic stenosis was found in higher mean proportions only in the herding, sporting, and working AKC groups. When assessing the disorder among the haplotype allele sharing groups, mastiff-like, retriever, and working dog 2 groups displayed higher proportions than those of the mixed-breed group. As noted above, the assignment of the German shepherd dog to the working dog 2 and the separate categories for retrievers and spaniels in the haplotype groupings may account for the difference observed between the AKC and haplotype groups. This would suggest that most breeds comprising the herding group are not at higher risk for aortic stenosis but the German shepherd is more prone which is consistent with the literature [16–18]. The present study also demonstrated that retrievers, but not spaniels, and mastiff-like dogs are more likely to present with aortic stenosis. Reports in the literature show that breeds defined by haplotype to be retrievers (Newfoundlands) and mastiff-like (boxers and the Dogue de Bordeaux) have a genetic predisposition for aortic stenosis [19–21] also corroborating the current findings.

Early onset cataracts showed a statistically greater probability of being found in two of the AKC breed groupings, the non-sporting and sporting groups. In the haplotype groupings, the retrievers and spaniels both showed a significant predisposition for cataracts as did the mastiff-like and working dog 1 groups. Epilepsy was more prevalent within the herding, hound, and sporting, particularly the spaniel breeds, groups. The risk for portosystemic shunt was only seen in the terrier and toy breed AKC groups and in the terrier, toy, and working dog 1 haplotype groups. The Havanese breed, classified as an AKC toy, is categorized in the haplotype working dog 1 group likely accounting for increased risk in that category.

Elevated risk of elbow dysplasia relative to the mixed-breed population was also limited to particular subsets of the purebred population: herding, sporting, and working AKC groups. Similar to the situation for aortic stenosis, when assessing the data for the haplotype share groupings, only retrievers and working dog 2 groups exhibited increased risk. This suggests that the breed contributing to the increased risk in the AKC herding group is the German shepherd dog, a breed having an elbow dysplasia prevalence of ~19 % (www.offa.org/stats_ed.html, accessed 07/14/2014). Retriever breeds account for the risk in the AKC sporting group. In contrast, mastiff-like breeds which may be expected to be predisposed to the condition did not show an increased risk for elbow dysplasia over that of the mixed-breed population. However, the Bernese mountain dog, the Newfoundland, and the Rottweiler breeds, having the highest prevalence for elbow dysplasia in this data set [6], are classified as retrievers by haplotype allele sharing. Golden retrievers, Labrador retrievers, German shepherds, Bernese mountain dogs, and Rottweilers are all known to be predisposed to elbow dysplasia [22]. The predisposition of these breeds for the inherited disorder further supports the common ancestry of the disorder shown by the association with breeds sharing alleles although environmental factors such as diet or exercise or enhanced screening by owners for these breeds also contribute to the prevalence reported. The diagnosis of elbow dysplasia represents a composite of presenting conditions, including humeral head osteochondrosis, fragmented coronoid process, and ununited anconeal process. Although the resolution of the diagnosis of elbow dysplasia in the present study does not allow attribution of individual conditions to breeds as done by LaFond et al. [23], the breed groupings at risk described above do mirror their findings.

Gastric dilatation volvulus (bloat), associated with particular breeds and also with large mixed-breed dogs [24], was restricted to only a few purebred subsets suggesting a common ancestry or a morphological component representing selection for similar phenotypic characteristics of breed standards to the expression of the condition. Breeds characteristically prone to GDV are large and giant breeds, particularly those with deep thoracic cavities [25]. Predisposition for GDV was seen in the AKC non-sporting and working groups and the working dog 1 and 2 groups for the haplotype categories. Standard poodles, acknowledged to have a breed predisposition for GDV [25], may account for the risk in the AKC non-sporting group because they are classified in the working dog 1 haplotype group.

Other disorders, such as atopy/allergic dermatitis, hypothyroidism, and IVDD, were more pervasive across the purebred population (Tables 2 and 3). For some disorders, such as hypothyroidism, purebred dogs may be more likely diagnosed with the condition due to pre-breeding health screens that are rarely done in the mixed-breed population.

For other disorders, although the study was designed to account for potential influences of weight on prevalence of disorders by comparing purebreds to mixed-breeds that were matched for weight, some disorders may reflect structural differences. Individual dogs may have equivalent weight but have significantly different morphologies. A preference for a particular morphology designated by a breed standard may contribute to a greater prevalence for purebreds, as in the case for IVDD. This concept is supported by the observation that the terrier group had lower risk when compared to the mixed-breed population. Terrier breeds generally emphasize a “square” outline with a short back. That can be contrasted with breeds more prone to IVDD which tend to have a significantly increased body length to height ratio [26]. The mixed-breed population would not have experienced selection toward increased body length to height ratio and therefore have less susceptibility to IVDD. Notably though, the overall prevalence within the mixed-breed population exceeded 4 % possibly reflecting the breeds at risk to IVDD contributing to the ancestry of a mixed-breed dog.

Study limitations: Notwithstanding the evaluation of ~ 90,000 dogs, a limitation to the study is that the data was derived from a regional referral teaching hospital. Particular purebred dogs of breeds having known predispositions for some conditions may have been seen at a greater frequency than that typically seen at a private veterinary hospital. Such a bias would inflate the prevalence of conditions in the study’s purebred dog population. However, many clients in the region use the teaching hospital as their regular clinic, and the relative proportion of purebred to mixed-breed clients seen during the study period reflect the United States national average [27] suggesting that this may not be a sizable limitation. Disorders that require more extensive diagnostic procedures may also inflate the risk determined because motivation to fully diagnose a condition may differ between owners of purebred and mixed-breed dog owners.

An additional limitation is that for some groupings, a condition may be rare or the number of dogs classified in a particular group was not frequently seen at the teaching hospital leading to imprecise risk estimates. For example, dilated cardiomyopathy was statistically more prevalent in the sight hound category yet had extremely broad confidence intervals reflecting that the sight hound group had the fewest number of dogs seen. Excessive odds ratio (OR) values should be interpreted with caution because though increased risk may exist, the magnitude of that risk may be lower than the value computed. For most breed groupings and disorders, there was sufficient power of analysis to reliably detect significant differences between the breed group and the mixed-breed population. For some disorders, certain breed groupings had a scarcity of affected dogs, such as for portosystemic shunt in retrievers. A larger sample of hospital records may mitigate this limitation. However, the low prevalence may reflect that the group has undergone concerted selection against the disorder or the condition does not have a testing scheme in place; a different study design would be needed to distinguish the latter. Similarly, the inability to determine risk for individual breeds reflects insufficient representation of every individual breed thereby preventing meaningful analyses of the contribution of individual breeds to genetic risk of a particular disorder. Finally, it would be ideal to have replicative data sets from multiple hospitals. Despite the limitations outlined, they do not invalidate the characterization of the contribution to the risk of specific inherited disorders by particular breed groupings or lineage.

Diversity and inherited disorders: Breeds, and lineages within breeds, are developed through concerted selected breeding often utilizing breeding schemes in which distantly or closely related individuals are bred to “fix” desirable morphological and behavioral traits permitting dogs to reliably pass on those traits to the next generation. As evidenced by some of the disorders in this study, different breeds likely shared distant ancestors if breeders sought similarities in function, morphology, and behavior. Frequent common ancestors in a pedigree have been implicated in reduced fitness and health [3, 28, 29] and loss of genetic diversity [30] although a study of dogs in the United Kingdom found that recent levels of inbreeding were less extensive than previously believed [31]. The relationship of inherited disorders and breed groupings in the present study supports the role of common ancestry to the contribution of risk.

Studies that have comprehensively assessed the genetic diversity of particular dog breeds have uncovered contradictions to the assumption that loss of diversity and inbreeding correlates with reduced health. One study reported that higher levels of inbreeding and reduced genetic diversity were associated with a reduction in the prevalence of hip dysplasia [32]. In a study that assessed the relationship of coefficients of inbreeding, and genomic microsatellite typing, to inherited disorders, the authors found “no clear correlation between the level of heterozygosity and the incidence or severity of the disease” [33]. Similarly, calculated inbreeding coefficients from pedigrees indicated substantial expected loss of genetic diversity but failed to demonstrate an association of diversity loss or recent inbreeding with breeds considered unhealthy [30]. Reduced genetic diversity of the major histocompatibility complex also does not correspond to lowered immune competence [34, 35].

A review by Wade [36] may provide insight into these seemingly contradictory findings. Using published genomic data, actual genetic diversity was compared to estimates derived from pedigree analyses; the pedigree analyses tended to underestimate the extent of genetic diversity [36]. Furthermore, various dog breeds retain approximately 87 % of the genomic diversity seen in the ancestral wolf [36] suggesting sufficient genetic diversity exists within breeds provided breeders do not homogenize the breeds.

The data presented here suggests that for some disorders associated with morphological selection, differential emphases in breed standards could reduce the incidence of inherited disorders in the purebred population [37]. However, some disorders, such as hip dysplasia, are more uniformly observed in the dog population as a whole [6]. Using hip dysplasia as an example, the physical, quadrapedal structure of a canine may increase the risk of hip dysplasia. For instance, Lawler et al. [38] report hip dysplasia in a red fox suggesting ancient hip dysplasia liability genes. Reducing the frequency of liability alleles widespread throughout the canine population will require careful selection schemes.

In a recent study, of the 20 most common conditions recorded in dogs seen at private veterinary hospitals in England [4], none would be considered as inherited by conventional standards. That study also compared prevalence of these common conditions between purebred and mixed-breed dogs and showed that purebred dogs had significantly higher prevalence only for otitis externa, obesity, and skin mass lesion. Furthermore prevalence was highly breed dependent, leading the authors to suggest that any breeding reforms with a goal to improve the health of dogs must consider conditions amenable to genetic improvement and do so on a “breed-by breed basis.” Thus, perhaps it is time to consider that all health conditions have some degree of inheritance and assessing lineage contributions may play a role in reducing incidence within all disorders.

## Conclusions

Despite the commonly held notion that mixed-breed dogs display fewer inherited disorders than purebred dogs [2–5], actual data suggests a more nuanced interpretation. Although some disorders are observed across the dog population as a whole [6], other disorders are observed with higher prevalence in the purebred dog population. The proportion of mixed-breed and subclassifications of purebred dogs for each of those conditions was determined to distinguish what specific backgrounds may contribute to the inherited conditions seen with higher prevalence in the purebred population or if in fact, purebreds as a whole were more at risk than the mixed-breed dogs. The present study illustrated that certain subpopulations of the purebred dog population were more likely to display certain conditions while other subpopulations were not statistically different than mixed-breed dogs in terms of disease prevalence.

The findings of the present study may shed light on the possible origin of certain inherited disorders in domestic dog evolution. Understanding inherited disorder origin and prevalence within subgroupings of the purebred population provides insight into what measures may be effective to reduce the incidence of particular conditions. The obvious differences in prevalence of the varied disorders indicates that a unilateral approach of mandating breeding reforms to improve the health of dogs is not the ideal approach; others have likewise suggested caution in applying breeding reforms that fail to consider the individual breeds [4, 29, 36]. Whether breeding reforms will mitigate inherited disorders in mixed-breeds will depend upon the locale. Some regions have a significant admixture of breeds in the mixed-breed population [39] whereas in other regions, the mixed-breed population may represent F_1_ crosses. Nevertheless, as most mixed-breed dogs have purebred ancestors, improvement of purebred genetic health may trickle down to mixed-breed dogs.

## Methods

Data: The data used in this study represent the interrogation of the electronic records of 90,004 unique dogs examined at the University of California—Davis Veterinary Medical Teaching Hospital from January 1, 1995 through January 1, 2010. The inherited disorders assessed, the criteria used to define dogs with and without particular disorders, and the breed designations have been detailed previously in Bellumori et al. [6]. Briefly, dogs were classified as having one of the 24 inherited disorders studied (hemangiosarcoma, lymphoma, mast cell tumor, osteosarcoma, aortic stenosis, dilated cardiomyopathy, hypertrophic cardiomyopathy, mitral valve dysplasia, patent ductus arteriosus, ventricular septal defect, hyperadrenocorticism, hypoadrenocorticism, hypothyroidism, elbow joint dysplasia, hip joint dysplasia, IVDD, patellar luxation, ruptured anterior cranial cruciate ligament, atopy or allergic dermatitis, GDV, cataracts in dogs 6 years or younger, epilepsy, lens luxation, and portosystemic liver shunt) only if the record included definitive confirmation of the condition by the veterinary medical teaching hospital staff or the referring veterinarian. Disorders having reliable diagnostics were selected to reflect different physiological systems with an expected impact on quality of life. Hospital controls from the same patient population were used in accordance with clinical research designs [40]. Specifically, dogs were classified as controls if none of the inherited conditions under study were diagnosed.

Because not all purebred breeds are designated into the AKC breed groupings, the number of dogs used in the analyses was 88,635. Purebred dogs of AKC breeds were categorized into one of the seven AKC breed group designations (*n* = 65,952): herding (*n* = 9724), hound (*n* = 5344), non-sporting (*n* = 6516), sporting (*n* = 18,940), terrier (*n* = 4885), toy (*n* = 9798), and working (*n* = 10,745) (Additional file 1: Table S1). Purebred dogs were also categorized into one of the 11 groupings based upon haplotype sharing as defined in Wayne and VonHoldt (2012) [14] (*n* = 55,353): ancient and spitz (*n* = 2946), herding (*n* = 4579), mastiff-like (*n* = 5548), retriever (*n* = 18,347), scent hounds (*n* = 3742), sight hounds (*n* = 761), small terriers (*n* = 2534), spaniels (*n* = 4315), toy (*n* = 5542), working dogs 1 (poodles and Havanese; *n* = 1923), and working dogs 2 (*n* = 5116) (Additional file 1: Table S1 and Additional file 2: Table S2). Mixed-breed dogs numbered 22,683.

Dogs were stratified by weight, age, sex, and breed category (AKC breed category or shared haplotype category) as previously described [6]. Mixed-breed dogs were also stratified by the same criteria.

Statistical analyses: For each disorder, appropriate population controls identified from the complete data file containing all unique dogs evaluated at the Veterinary Medical Teaching Hospital in the 15-year time frame were used. Because the total number of dogs lacking a given condition far exceeded the number of dogs with the condition, to create the control population against which the dogs with the condition were compared, it was necessary to randomly sample among the dogs lacking the condition.

Ten disorders studied exhibited a statistically significant elevated prevalence in the purebred population when compared to the mixed-breed population: aortic stenosis, atopy/allergic dermatitis, GDV, early onset cataracts, dilated cardiomyopathy, elbow dysplasia, epilepsy, hypothyroidism, IVDD, and portosystemic shunt [6]. The number of dogs not having the disorder was greater than the number of dogs with the disorder. Thus individual dogs having one of these ten disorders were matched to a randomly selected dog from the control group having the same weight, sex, and age classification. For each disorder evaluated, the sampled data set was used to estimate the proportion of each purebred breed category with the disorder relative to the proportion of mixed-breed dogs with the disorder. For each condition, the random sampling from the control dogs was repeated [6]. Data sets were created that had identical characteristics between control dogs and those with disorders with the exception of disease status.

All analyses were conducted as previously described [6]. Specifically, the sampling was repeated 50 times for each inherited disorder to generate a robust odds ratio (OR), its 95 % confidence interval (CI), and the mean *P* value testing this ratio against the null hypothesis of 1.0 to assess the disease risk for each purebred category relative to the mixed-breed dog population. Briefly, a logit link function with the model terms including the class variables of age, weight, sex and breed group was applied to the binomial disorder status. In each of the 50 samples, a new control set was developed. The *P* values presented and used to assess significant departures from an OR of 1.0 is the mean of the 50 individual values each computed from the randomly generated data sets. The re-sampling process used to generate the 50 data sets was done to minimize exposure to false positive declarations of significant departures from an OR of 1.0. Accordingly, any post-hoc adjustment of *P* values (e.g., Bonferroni adjustment) was unnecessary. The ORs generated from each disorder analyses of the 50 sets were averaged along with the lower and upper 95 % CIs and the associated *P* values.

## Additional files

Additional file 1: Table S1. Breeds categorized by AKC breed group and by haplotype allele sharing as per Wayne and VonHoldt [1].) Additional file 2: Table S2. Breeds categorized by haplotype allele sharing as per Wayne and VonHoldt (2012) with speculated designations denoted in red font based upon Parker et al. (2004).

## Abbreviations

AKC: American Kennel Club
CI: confidence interval
GDV: gastric dilatation volvulus
IVDD: intervertebral disk disease
OR: odds ratio
