# Supplementary material for: Ten inherited disorders in purebred dogs by functional breed groupings
Source: Canine Genet Epidemiol. 2015 Jul 11;2:9. doi: 10.1186/s40575-015-0021-x (PMC4579393; doi:10.1186/s40575-015-0021-x)
Supplement: Additional file 1: Table S1. — Breeds categorized by AKC breed group and by haplotype allele sharing as per Wayne and VonHoldt [1].) [file 40575_2015_21_MOESM1_ESM.docx]

Additional file 1: Table S1: Breeds categorized by AKC breed group and by haplotype allele sharing as per Wayne and VonHoldt [[1](#_ENREF_1)].

| \|  \| **Association Designation** \| \|  \| \| --- \| --- \| --- \| --- \| \| **Breed^A^** \| **AKC Breed Group** \| **Haplotype Sharing Group** \| **Number of dogs in breed** \| \| Australian Shepherd \| Herding \| Herding Dogs \| 1423 \| \| Border Collie \| Herding \| Herding Dogs \| 1223 \| \| Collie, Rough and Smooth \| Herding \| Herding Dogs \| 325 \| \| Old English Sheepdog \| Herding \| Herding Dogs \| 145 \| \| Shetland Sheepdog \| Herding \| Herding Dogs \| 876 \| \| Welsh Corgi, Cardigan \| Herding \| Herding Dogs \| 64 \| \| Welsh Corgi, Pembroke \| Herding \| Herding Dogs \| 523 \| \| Briard \| Herding \| Mastiff-like breeds \| 60 \| \| German Shepherd Dog \| Herding \| Working Dogs 2 \| 3614 \| \| Australian Cattle Dog \| Herding \|  \| 899 \| \| Bearded Collie \| Herding \|  \| 82 \| \| Beauceron \| Herding \|  \| 12 \| \| Belgian Malinois \| Herding \|  \| 132 \| \| Belgian Sheepdog \| Herding \|  \| 66 \| \| Belgian Tervuren \| Herding \|  \| 70 \| \| Bouvier Des Flandres \| Herding \|  \| 168 \| \| Canaan Dog \| Herding \|  \| 7 \| \| Entlebucher Mountain Dog \| Herding \|  \| 7 \| \| Polish Lowland Sheepdog \| Herding \|  \| 4 \| \| Puli \| Herding \|  \| 24 \| \| Afghan Hound \| Hound \| Ancient & Spitz Dogs \| 75 \| \| Basenji \| Hound \| Ancient & Spitz Dogs \| 109 \| \| Saluki \| Hound \| Ancient & Spitz Dogs \| 85 \| \| Basset Hound \| Hound \| Scent Hounds \| 449 \| \| Beagle \| Hound \| Scent Hounds \| 784 \| \| Bloodhound \| Hound \| Scent Hounds \| 59 \| \| Dachshund \| Hound \| Scent Hounds \| 2397 \| \| Petit Basset Griffon Vendeen \| Hound \| Scent Hounds \| 53 \| \| Borzoi \| Hound \| Sight hounds \| 83 \| \| Greyhound \| Hound \| Sight hounds \| 235 \| \| Irish Wolfhound \| Hound \| Sight hounds \| 148 \| \| Scottish Deerhound \| Hound \| Sight hounds \| 27 \| \| Whippet \| Hound \| Sight hounds \| 168 \| \| American Foxhound \| Hound \|  \| 19 \| \| Black And Tan Coonhound \| Hound \|  \| 11 \| \| Bluetickhound \| Hound \|  \| 17 \| \| Coonhound \| Hound \|  \| 48 \| \| English Foxhound \| Hound \|  \| 3 \| \| Harrier \| Hound \|  \| 4 \| \| Ibizan Hound \| Hound \|  \| 9 \| \| Norwegian Elkhound \| Hound \|  \| 82 \| \| Otterhound \| Hound \|  \| 5 \| \| Pharaoh Hound \| Hound \|  \| 7 \| \| Plott Hound \| Hound \|  \| 16 \| \| Redbone Coonhound \| Hound \|  \| 23 \| \| Rhodesian Ridgeback \| Hound \|  \| 428 \| \| American Eskimo Dog \| Non-Sporting \| Ancient & Spitz Dogs \| 247 \| \| Chow Chow \| Non-Sporting \| Ancient & Spitz Dogs \| 429 \| \| Sharpei \| Non-Sporting \| Ancient & Spitz Dogs \| 442 \| \| Boston Terrier \| Non-Sporting \| Mastiff-like breeds \| 617 \| \| Bulldog (English and French) \| Non-Sporting \| Mastiff-like breeds \| 983 \| \| Poodle, Standard \| Non-Sporting \| Working Dogs 1 \| 1128 \| \| Bichon Frise \| Non-Sporting \|  \| 555 \| \| Dalmatian \| Non-Sporting \|  \| 804 \| \| Finnish Spitz \| Non-Sporting \|  \| 21 \| \| Keeshond \| Non-Sporting \|  \| 166 \| \| Lhasa Apso \| Non-Sporting \|  \| 619 \| \| Schipperke \| Non-Sporting \|  \| 146 \| \| Shiba Inu \| Non-Sporting \|  \| 118 \| \| Tibetan Spaniel \| Non-Sporting \|  \| 19 \| \| Tibetan Terrier \| Non-Sporting \|  \| 222 \| \| Flatcoated Retriever \| Sporting \| Retriever \| 129 \| \| Golden Retriever \| Sporting \| Retriever \| 4678 \| \| Labrador Retriever \| Sporting \| Retriever \| 8655 \| \| Brittany Spaniel \| Sporting \| Spaniels \| 407 \| \| Cocker Spaniel, American and English \| Sporting \| Spaniels \| 2122 \| \| German Short Haired Pointer \| Sporting \| Spaniels \| 652 \| \| Irish Water Spaniel \| Sporting \| Spaniels \| 10 \| \| Springer Spaniel, English \| Sporting \| Spaniels \| 776 \| \| American Water Spaniel \| Sporting \|  \| 12 \| \| Boykin Spaniel \| Sporting \|  \| 5 \| \| Chesapeake Bay Retriever \| Sporting \|  \| 285 \| \| Clumber Spaniel \| Sporting \|  \| 23 \| \| Curly Coated Retriever \| Sporting \|  \| 14 \| \| English Pointer \| Sporting \|  \| 132 \| \| English Setter \| Sporting \|  \| 119 \| \| German Wire Haired Pointer \| Sporting \|  \| 97 \| \| Gordon Setter \| Sporting \|  \| 81 \| \| Irish Setter \| Sporting \|  \| 117 \| \| Springer Spaniel, Welsh \| Sporting \|  \| 28 \| \| Sussex Spaniel \| Sporting \|  \| 2 \| \| Vizsla \| Sporting \|  \| 251 \| \| Weimaraner \| Sporting \|  \| 322 \| \| Wirehaired Pointing Griffon \| Sporting \|  \| 23 \| \| Jack Russell Terrier \| Terrier \| Mastiff-like breeds \| 1022 \| \| Staffordshire Terrier \| Terrier \| Mastiff-like breeds \| 446 \| \| Australian Terrier \| Terrier \| Small terriers \| 59 \| \| Cairn Terrier \| Terrier \| Small terriers \| 217 \| \| Norwich Terrier \| Terrier \| Small terriers \| 54 \| \| Scottish Terrier \| Terrier \| Small terriers \| 269 \| \| West Highland White Terrier \| Terrier \| Small terriers \| 536 \| \| Bullterrier \| Terrier \|  \| 195 \| \| Airedale Terrier \| Terrier \|  \| 288 \| \| Bedlington Terrier \| Terrier \|  \| 16 \| \| Border Terrier \| Terrier \|  \| 101 \| \| Dandie Dinmont Terrier \| Terrier \|  \| 10 \| \| Fox Terrier \| Terrier \|  \| 298 \| \| Irish Terrier \| Terrier \|  \| 32 \| \| Kerryblue Terrier \| Terrier \|  \| 48 \| \| Lakeland Terrier \| Terrier \|  \| 20 \| \| Norfolk Terrier \| Terrier \|  \| 26 \| \| Schnauzer, Miniature \| Terrier \|  \| 1008 \| \| Sealyham Terrier \| Terrier \|  \| 14 \| \| Skye Terrier \| Terrier \|  \| 12 \| \| Soft Coated Wheaten Terrier \| Terrier \|  \| 149 \| \| Welsh Terrier \| Terrier \|  \| 65 \| \| Italian Greyhound \| Toy \| Sight hounds \| 100 \| \| Yorkshire Terrier \| Toy \| Small terriers \| 1399 \| \| Cavalier King Charles Spaniel \| Toy \| Spaniels \| 348 \| \| Brussels Griffon \| Toy \| Toy Dogs \| 39 \| \| Chihuahua \| Toy \| Toy Dogs \| 1723 \| \| Miniature Pinscher \| Toy \| Toy Dogs \| 424 \| \| Papillon \| Toy \| Toy Dogs \| 147 \| \| Pekingese \| Toy \| Toy Dogs \| 306 \| \| Pomeranian \| Toy \| Toy Dogs \| 836 \| \| Pug \| Toy \| Toy Dogs \| 970 \| \| Shih Tzu \| Toy \| Toy Dogs \| 1097 \| \| Havanese \| Toy \| Working Dogs 1 \| 92 \| \| Poodle, Toy \| Toy \| Working Dogs 1 \| 703 \| \| Poodle, Miniature \| Toy \|  \| 537 \| \| Affenpinscher \| Toy \|  \| 9 \| \| Chinese Crested \| Toy \|  \| 47 \| \| Fox Terrier, Toy \| Toy \|  \| 91 \| \| Japanese Chin \| Toy \|  \| 43 \| \| Maltese \| Toy \|  \| 715 \| \| Manchester Terrier \| Toy \|  \| 41 \| \| Silky Terrier \| Toy \|  \| 123 \| \| Toy Manchester Terrier \| Toy \|  \| 8 \| \| Akita \| Working \| Ancient & Spitz Dogs \| 422 \| \| Alaskan Malamute \| Working \| Ancient & Spitz Dogs \| 335 \| \| Samoyed \| Working \| Ancient & Spitz Dogs \| 226 \| \| Siberian Husky \| Working \| Ancient & Spitz Dogs \| 576 \| \| Boxer \| Working \| Mastiff-like breeds \| 1714 \| \| Bullmastiff \| Working \| Mastiff-like breeds \| 263 \| \| Mastiff \| Working \| Mastiff-like breeds \| 443 \| \| Bernese Mountain Dog \| Working \| Retriever \| 460 \| \| Great Dane \| Working \| Retriever \| 644 \| \| Newfoundland \| Working \| Retriever \| 603 \| \| Rottweiler \| Working \| Retriever \| 2965 \| \| Saint Bernard \| Working \| Retriever \| 213 \| \| Doberman Pinscher \| Working \| Working Dogs 2 \| 984 \| \| Portuguese Water Dog \| Working \| Working Dogs 2 \| 120 \| \| Schnauzer (Giant and Standard) \| Working \| Working Dogs 2 \| 398 \| \| Anatolian Shepherd Dog \| Working \|  \| 37 \| \| Dogue De Bordeaux \| Working \|  \| 26 \| \| Great Pyrenees \| Working \|  \| 175 \| \| Komondor \| Working \|  \| 9 \| \| Kuvasz \| Working \|  \| 16 \| \| Leonberger \| Working \|  \| 27 \| \| Neapolitan Mastiff \| Working \|  \| 46 \| \| Swiss Mountain Dog \| Working \|  \| 43 \| |  |
| --- | --- | --- | --- | --- | --- | --- | --- | --- | --- | --- | --- | --- | --- | --- | --- | --- | --- | --- | --- | --- | --- | --- | --- | --- | --- | --- | --- | --- | --- | --- | --- | --- | --- | --- | --- | --- | --- | --- | --- | --- | --- | --- | --- | --- | --- | --- | --- | --- | --- | --- | --- | --- | --- | --- | --- | --- | --- | --- | --- | --- | --- | --- | --- | --- | --- | --- | --- | --- | --- | --- | --- | --- | --- | --- | --- | --- | --- | --- | --- | --- | --- | --- | --- | --- | --- | --- | --- | --- | --- | --- | --- | --- | --- | --- | --- | --- | --- | --- | --- | --- | --- | --- | --- | --- | --- | --- | --- | --- | --- | --- | --- | --- | --- | --- | --- | --- | --- | --- | --- | --- | --- | --- | --- | --- | --- | --- | --- | --- | --- | --- | --- | --- | --- | --- | --- | --- | --- | --- | --- | --- | --- | --- | --- | --- | --- | --- | --- | --- | --- | --- | --- | --- | --- | --- | --- | --- | --- | --- | --- | --- | --- | --- | --- | --- | --- | --- | --- | --- | --- | --- | --- | --- | --- | --- | --- | --- | --- | --- | --- | --- | --- | --- | --- | --- | --- | --- | --- | --- | --- | --- | --- | --- | --- | --- | --- | --- | --- | --- | --- | --- | --- | --- | --- | --- | --- | --- | --- | --- | --- | --- | --- | --- | --- | --- | --- | --- | --- | --- | --- | --- | --- | --- | --- | --- | --- | --- | --- | --- | --- | --- | --- | --- | --- | --- | --- | --- | --- | --- | --- | --- | --- | --- | --- | --- | --- | --- | --- | --- | --- | --- | --- | --- | --- | --- | --- | --- | --- | --- | --- | --- | --- | --- | --- | --- | --- | --- | --- | --- | --- | --- | --- | --- | --- | --- | --- | --- | --- | --- | --- | --- | --- | --- | --- | --- | --- | --- | --- | --- | --- | --- | --- | --- | --- | --- | --- | --- | --- | --- | --- | --- | --- | --- | --- | --- | --- | --- | --- | --- | --- | --- | --- | --- | --- | --- | --- | --- | --- | --- | --- | --- | --- | --- | --- | --- | --- | --- | --- | --- | --- | --- | --- | --- | --- | --- | --- | --- | --- | --- | --- | --- | --- | --- | --- | --- | --- | --- | --- | --- | --- | --- | --- | --- | --- | --- | --- | --- | --- | --- | --- | --- | --- | --- | --- | --- | --- | --- | --- | --- | --- | --- | --- | --- | --- | --- | --- | --- | --- | --- | --- | --- | --- | --- | --- | --- | --- | --- | --- | --- | --- | --- | --- | --- | --- | --- | --- | --- | --- | --- | --- | --- | --- | --- | --- | --- | --- | --- | --- | --- | --- | --- | --- | --- | --- | --- | --- | --- | --- | --- | --- | --- | --- | --- | --- | --- | --- | --- | --- | --- | --- | --- | --- | --- | --- | --- | --- | --- | --- | --- | --- | --- | --- | --- | --- | --- | --- | --- | --- | --- | --- | --- | --- | --- | --- | --- | --- | --- | --- | --- | --- | --- | --- | --- | --- | --- | --- | --- | --- | --- | --- | --- | --- | --- | --- | --- | --- | --- | --- | --- | --- | --- | --- | --- | --- | --- | --- | --- | --- | --- | --- | --- | --- | --- | --- | --- | --- | --- | --- | --- | --- | --- | --- | --- | --- | --- | --- | --- | --- | --- | --- | --- | --- | --- | --- | --- | --- | --- | --- | --- | --- | --- | --- | --- | --- | --- | --- | --- | --- | --- | --- | --- | --- | --- | --- | --- | --- | --- | --- | --- | --- | --- | --- | --- | --- | --- | --- | --- | --- | --- | --- | --- | --- | --- | --- | --- | --- | --- | --- | --- | --- | --- | --- | --- | --- | --- | --- | --- | --- | --- | --- | --- | --- | --- | --- | --- | --- | --- | --- | --- | --- | --- | --- | --- | --- | --- | --- | --- | --- | --- | --- | --- | --- | --- | --- | --- | --- | --- | --- | --- | --- | --- | --- | --- | --- | --- | --- | --- | --- | --- | --- | --- | --- | --- | --- |
|  |  |

^A^ Breeds highlighted in yellow differ in assignment between the two association designations.

1. Wayne RK, VonHoldt BM: **Evolutionary genomics of dog domestication.** *Mammalian Genome* 2012, **23:**3-18.
