# Supplementary material for: Ten inherited disorders in purebred dogs by functional breed groupings
Source: Canine Genet Epidemiol. 2015 Jul 11;2:9. doi: 10.1186/s40575-015-0021-x (PMC4579393; doi:10.1186/s40575-015-0021-x)
Supplement: Additional file 2: Table S2. — Breeds categorized by haplotype allele sharing as per Wayne and VonHoldt (2012) with speculated designations denoted in red font based upon Parker et al. (2004). [file 40575_2015_21_MOESM2_ESM.docx]

Additional file 2: Table S2: Breeds categorized by haplotype allele sharing as per Wayne and VonHoldt (2012) with speculated designations denoted in red font based upon Parker et al. (2004).

|  | **Designation** | |  |
| --- | --- | --- | --- |
| **Breed** | **AKC Breed Group** | **Haplotype Sharing Group**  **(designations in red are speculative)** | **Number of dogs of each breed** |
| Australian Shepherd | Herding | Herding Dogs | 1423 |
| Border Collie | Herding | Herding Dogs | 1223 |
| Collie, Rough and Smooth | Herding | Herding Dogs | 325 |
| Old English Sheepdog | Herding | Herding Dogs | 145 |
| Shetland Sheepdog | Herding | Herding Dogs | 876 |
| Welsh Corgi, Cardigan | Herding | Herding Dogs | 64 |
| Welsh Corgi, Pembroke | Herding | Herding Dogs | 523 |
| Briard | Herding | Mastiff-like breeds | 60 |
| German Shepherd Dog | Herding | Working Dogs 2 | 3614 |
| Australian Cattle Dog | Herding | Herding Dogs | 899 |
| Bearded Collie | Herding | Herding Dogs | 82 |
| Beauceron | Herding | Working Dogs 2 | 12 |
| Belgian Malinois | Herding | Herding Dogs | 132 |
| Belgian Sheepdog | Herding | Herding Dogs | 66 |
| Belgian Tervuren | Herding | Herding Dogs | 70 |
| Bouvier Des Flandres | Herding | Working Dogs 2 | 168 |
| Canaan Dog | Herding | Ancient & Spitz Dogs | 7 |
| Entlebucher Mountain Dog | Herding | Herding Dogs | 7 |
| Polish Lowland Sheepdog | Herding | Herding Dogs | 4 |
| Puli | Herding | Herding Dogs | 24 |
| Afghan Hound | Hound | Ancient & Spitz Dogs | 75 |
| Basenji | Hound | Ancient & Spitz Dogs | 109 |
| Saluki | Hound | Ancient & Spitz Dogs | 85 |
| Basset Hound | Hound | Scent Hounds | 449 |
| Beagle | Hound | Scent Hounds | 784 |
| Bloodhound | Hound | Scent Hounds | 59 |
| Dachshund | Hound | Scent Hounds | 2397 |
| Petit Basset Griffon Vendeen | Hound | Scent Hounds | 53 |
| Borzoi | Hound | Sight hounds | 83 |
| Greyhound | Hound | Sight hounds | 235 |
| Irish Wolfhound | Hound | Sight hounds | 148 |
| Scottish Deerhound | Hound | Sight hounds | 27 |
| Whippet | Hound | Sight hounds | 168 |
| American Foxhound | Hound | Scent Hounds | 19 |
| Black And Tan Coonhound | Hound | Scent Hounds | 11 |
| Bluetick Hound | Hound | Scent Hounds | 17 |
| Coonhound | Hound | Scent Hounds | 48 |
| English Foxhound | Hound | Scent Hounds | 3 |
| Harrier | Hound | Scent Hounds | 4 |
| Ibizan Hound | Hound | Sight hounds | 9 |
| Norwegian Elkhound | Hound | Spaniels | 82 |
| Otterhound | Hound | Scent Hounds | 5 |
| Pharaoh Hound | Hound | Scent Hounds | 7 |
| Plott Hound | Hound | Scent Hounds | 16 |
| Redbone Coonhound | Hound | Scent Hounds | 23 |
| Rhodesian Ridgeback | Hound | Retriever | 428 |
| American Eskimo Dog | Non-Sporting | Ancient & Spitz Dogs | 247 |
| Chow Chow | Non-Sporting | Ancient & Spitz Dogs | 429 |
| Sharpei | Non-Sporting | Ancient & Spitz Dogs | 442 |
| Boston Terrier | Non-Sporting | Mastiff-like breeds | 617 |
| Bulldog (English and French) | Non-Sporting | Mastiff-like breeds | 983 |
| Poodle, Standard | Non-Sporting | Working Dogs 1 | 1128 |
| Bichon Frise | Non-Sporting | Toy Dogs | 555 |
| Dalmatian | Non-Sporting | Retriever | 804 |
| Finnish Spitz | Non-Sporting | Ancient & Spitz Dogs | 21 |
| Keeshond | Non-Sporting | Spaniels | 166 |
| Lhasa Apso | Non-Sporting | Toy Dogs | 619 |
| Schipperke | Non-Sporting | Working Dogs 1 | 146 |
| Shiba Inu | Non-Sporting | Ancient & Spitz Dogs | 118 |
| Tibetan Spaniel | Non-Sporting | Spaniels | 19 |
| Tibetan Terrier | Non-Sporting | Small terriers | 222 |
| Flatcoated Retriever | Sporting | Retriever | 129 |
| Golden Retriever | Sporting | Retriever | 4678 |
| Labrador Retriever | Sporting | Retriever | 8655 |
| Brittany Spaniel | Sporting | Spaniels | 407 |
| Cocker Spaniel, American and English | Sporting | Spaniels | 2122 |
| German Short Haired Pointer | Sporting | Spaniels | 652 |
| Irish Water Spaniel | Sporting | Spaniels | 10 |
| Springer Spaniel, English | Sporting | Spaniels | 776 |
| American Water Spaniel | Sporting | Spaniels | 12 |
| Boykin Spaniel | Sporting | Spaniels | 5 |
| Chesapeake Bay Retriever | Sporting | Retriever | 285 |
| Clumber Spaniel | Sporting | Spaniels | 23 |
| Curly Coated Retriever | Sporting | Retriever | 14 |
| English Pointer | Sporting | Spaniels | 132 |
| English Setter | Sporting | Spaniels | 119 |
| German Wire Haired Pointer | Sporting | Spaniels | 97 |
| Gordon Setter | Sporting | Spaniels | 81 |
| Irish Setter | Sporting | Spaniels | 117 |
| Springer Spaniel, Welsh | Sporting | Spaniels | 28 |
| Sussex Spaniel | Sporting | Spaniels | 2 |
| Vizsla | Sporting | Retriever | 251 |
| Weimaraner | Sporting | Retriever | 322 |
| Wirehaired Pointing Griffon | Sporting | Spaniels | 23 |
| Jack Russell Terrier | Terrier | Mastiff-like breeds | 1022 |
| Staffordshire Terrier | Terrier | Mastiff-like breeds | 446 |
| Australian Terrier | Terrier | Small terriers | 59 |
| Cairn Terrier | Terrier | Small terriers | 217 |
| Norwich Terrier | Terrier | Small terriers | 54 |
| Scottish Terrier | Terrier | Small terriers | 269 |
| West Highland White Terrier | Terrier | Small terriers | 536 |
| Bullterrier | Terrier | Mastiff-like breeds | 195 |
| Airedale Terrier | Terrier | Small terriers | 288 |
| Bedlington Terrier | Terrier | Small terriers | 16 |
| Border Terrier | Terrier | Small terriers | 101 |
| Dandie Dinmont Terrier | Terrier | Small terriers | 10 |
| Fox Terrier | Terrier | Small terriers | 298 |
| Irish Terrier | Terrier | Small terriers | 32 |
| Kerryblue Terrier | Terrier | Small terriers | 48 |
| Lakeland Terrier | Terrier | Small terriers | 20 |
| Norfolk Terrier | Terrier | Small terriers | 26 |
| Schnauzer, Miniature | Terrier | Small terriers | 1008 |
| Sealyham Terrier | Terrier | Small terriers | 14 |
| Skye Terrier | Terrier | Small terriers | 12 |
| Soft Coated Wheaten Terrier | Terrier | Small terriers | 149 |
| Welsh Terrier | Terrier | Small terriers | 65 |
| Italian Greyhound | Toy | Sight hounds | 100 |
| Yorkshire Terrier | Toy | Small terriers | 1399 |
| Cavalier King Charles Spaniel | Toy | Spaniels | 348 |
| Brussels Griffon | Toy | Toy Dogs | 39 |
| Chihuahua | Toy | Toy Dogs | 1723 |
| Miniature Pinscher | Toy | Toy Dogs | 424 |
| Papillon | Toy | Toy Dogs | 147 |
| Pekingese | Toy | Toy Dogs | 306 |
| Pomeranian | Toy | Toy Dogs | 836 |
| Pug | Toy | Toy Dogs | 970 |
| Shih Tzu | Toy | Toy Dogs | 1097 |
| Havanese | Toy | Working Dogs 1 | 92 |
| Poodle, Toy | Toy | Working Dogs 1 | 703 |
| Poodle, Miniature | Toy | Working Dogs 1 | 537 |
| Affenpinscher | Toy | Spaniels | 9 |
| Chinese Crested | Toy | Ancient & Spitz Dogs | 47 |
| Fox Terrier, Toy | Toy | Small terriers | 91 |
| Japanese Chin | Toy | Toy Dogs | 43 |
| Maltese | Toy | Toy Dogs | 715 |
| Manchester Terrier | Toy | Spaniels | 41 |
| Silky Terrier | Toy | Small terriers | 123 |
| Toy Manchester Terrier | Toy | Small terriers | 8 |
| Akita | Working | Ancient & Spitz Dogs | 422 |
| Alaskan Malamute | Working | Ancient & Spitz Dogs | 335 |
| Samoyed | Working | Ancient & Spitz Dogs | 226 |
| Siberian Husky | Working | Ancient & Spitz Dogs | 576 |
| Boxer | Working | Mastiff-like breeds | 1714 |
| Bullmastiff | Working | Mastiff-like breeds | 263 |
| Mastiff | Working | Mastiff-like breeds | 443 |
| Bernese Mountain Dog | Working | Retriever | 460 |
| Great Dane | Working | Retriever | 644 |
| Newfoundland | Working | Retriever | 603 |
| Rottweiler | Working | Retriever | 2965 |
| Saint Bernard | Working | Retriever | 213 |
| Doberman Pinscher | Working | Working Dogs 2 | 984 |
| Portuguese Water Dog | Working | Working Dogs 2 | 120 |
| Schnauzer (Giant and Standard) | Working | Small terriers | 398 |
| Anatolian Shepherd Dog | Working | Working Dogs 2 | 37 |
| Dogue De Bordeaux | Working | Working Dogs 2 | 26 |
| Great Pyrenees | Working | Retriever | 175 |
| Komondor | Working | Working Dogs 1 | 9 |
| Kuvasz | Working | Working Dogs 1 | 16 |
| Leonberger | Working | Retriever | 27 |
| Neapolitan Mastiff | Working | Mastiff-like breeds | 46 |
| Swiss Mountain Dog | Working | Retriever | 43 |

Parker HG, Kim LV, Sutter NB, Carlson S, Lorentzen TD, Malek TB, Johnson GS, DeFrance HB, Ostrander EA, Kruglyak L.: **Genetic structure of the purebred domestic dog.** *Science* 2004, 304:1160-4.

Wayne RK, VonHoldt BM: **Evolutionary genomics of dog domestication.** *Mammalian Genome* 2012, **23:**3-18.
